# Testing the Deliberate Practice Theory: Does Practice Reduce the Heritability of Musical Expertise?

**DOI:** 10.3390/jintelligence12090087

**Published:** 2024-09-08

**Authors:** Miriam A. Mosing, Karin J. H. Verweij, David Z. Hambrick, Nancy L. Pedersen, Fredrik Ullén

**Affiliations:** 1 Department of Cognitive Neuropsychology, Max Planck Institute for Empirical Aesthetics, Grüneburgweg 14, 60322 Frankfurt am Main, Germany; fredrik.ullen@ae.mpg.de; 2 Department of Neuroscience, Karolinska Institutet, SE-171 77 Stockholm, Sweden; 3 Department of Medical Epidemiology and Biostatistics, Karolinska Institutet, SE-171 77 Stockholm, Sweden; 4 Department of Psychiatry, Amsterdam UMC, University of Amsterdam, Meibergdreef 9, 1105 AZ Amsterdam, The Netherlands; 5 Department of Psychology, Michigan State University, East Lansing, MI 48824, USA

**Keywords:** skills, training, music, expertise, deliberate practice theory, behaviour genetics

## Abstract

The deliberate practice (DP) theory claims that expertise essentially reflects the accumulated amount of deliberate practice, and that with sufficient practice, genetic influences on expertise will be bypassed. Thus, a core prediction of the DP theory is that genetic effects on performance decrease as a function of practice. Here, we test this prediction using music as a model domain. Musical expertise (measured with a musical auditory discrimination test) and lifetime practice hours were determined in 6471 twins including 1302 complete twin pairs. We fitted a bivariate Cholesky decomposition with practice hours as a moderator to determine to what extent genetic and environmental influences on musical expertise are influenced by practice hours. On average, 50% of individual differences in musical expertise were due to genetic influences, whereas shared environmental and residual influences each explained about 25%. Importantly, music practice significantly moderated these estimates. Variation in musical expertise decreased with more practice hours due to decreased shared environmental and residual variance. In contrast, the overall genetic component was unaffected by the number of practice hours. Consequently, the relative genetic contribution (heritability) increased with more practice hours. These findings are in contrast with predictions from the DP theory and suggest that genetic predisposition remains important for musical expertise even after prolonged practice.

## 1. Introduction

Consistent with everyday observation, substantial phenotypic variation in humans has been observed across most domains, with some individuals being far more skilled than others in a given field. Some people are simply (much) better at complex tasks such as music, sports, and games than others. The question of whether this variability is more a reflection of nature or nurture—whether experts are ‘born’ or ‘made’—is one of the most intensely debated questions in differential psychology (Detterman and Ruthsatz 1999; Ericsson et al. 2005; Howe et al. 1998; Ruthsatz et al. 2008; Ullén et al. 2016).

A paradigm that has been particularly influential in expertise research in recent decades is the deliberate practice (DP) theory developed by Ericsson and colleagues (Ericsson and Smith 1991; Ericsson and Ward 2007; Feltovich et al. 2006). The DP theory emphasises the importance of practice as an environmental determinant of skill and more specifically proposes that expertise largely reflects the accumulated amount of DP, defined as explicit, effortful, goal-directed activities that are specifically designed to improve performance in a domain (Ericsson 2006). Based on this idea, practice is something individuals can engage in to improve their performance and to ‘circumvent’ any initial genetic limitations in capacity, suggesting that it is not only important and necessary, but that it is sufficient to account for the majority of, if not all, differences in expertise (Ericsson 2014a; Ericsson et al. 1993, 2005; Ericsson and Ward 2007). As Ericsson (2007) stated, “the distinctive characteristics of elite performers are adaptations to extended and intense practice activities that selectively activate dormant genes that all healthy children’s DNA contain” (p. 4). And as he reiterated in his recent book, *Peak*, “If one follows these methods carefully and diligently [follows accepted training methods], one will almost surely become an expert” (Ericsson and Pool 2016).

Recently, the DP theory has been challenged by several lines of research findings (for recent comprehensive and critical reviews, see Hambrick et al. 2016; Ullén et al. 2016; Wesseldijk et al. 2023). A number of studies have applied twin designs to test predictions based on the DP theory. Some studies have estimated genetic and environmental influences on different types of expertise, finding substantial genetic influences. For example, Vinkhuyzen et al. (2009) reported heritability estimates ranging between 32% and 93% for self-rated aptitudes and talents in several different domains, including music, creative writing, arts, foreign language ability, chess and related games, mathematics, athletic skills, factual knowledge, and memory skills. Plomin et al. (2014) showed that genetic factors play a role in exceptional performance in reading (genes account for more than half of the difference between expert and normal readers), whereas shared environmental influences, such as growing up in the same family and attending the same school, only account for less than a fifth of the difference. A recent review on music and genetics showed the average heritabilities for music-related traits to be around 40%, increasing to 86% for exceptional musical talent. Taken together, these findings strongly suggest, contrary to the DP theory, that there is a genetic predisposition to acquire expertise, with some individuals simply being more talented than others.

Evidence from twin studies further indicates that genes influence not just expert performance but also the predisposition to practice. Two studies have reported substantial genetic influences on music practice, ranging between 38% and 70% (Hambrick and Tucker-Drob 2015; Mosing et al. 2014a), suggesting that the willingness to practice may be partly under genetic influence, with some individuals being more likely to engage and persist in the learning of a specific skill. This is in line with the findings of substantial and well-established genetic influences on educational attainment (Branigan et al. 2013; Cesarini and Visscher 2017).

The DP theory also makes clear predictions about the nature of phenotypic associations between practice and performance, stating that practice (as an environmental factor) causally influences performance and that variance in performance is largely due to differences in practice behaviour. While practice can undoubtedly improve performance, a few recent twin studies found that genetic mechanisms may contribute substantially to associations between practice and musical expertise. Mosing et al. (2014a) showed that genetic pleiotropy explained much of the association between music practice and musical ability and that the effect of music practice, either for musical ability or accuracy of motor timing (Ullén et al. 2015), was likely not causal, with more trained twins performing no better than their less trained genetically identical co-twins. 

A central claim of the DP theory is that the importance of genetic factors for skilled performance will decrease and ultimately approach zero with prolonged practice (e.g., Ericsson 2014b; Ericsson et al. 1993). An early twin study on short-term training of the rotary pursuit task indicated that the learning rate is partly heritable and that genetic influences on performance increased after three days of training (Fox et al. 1996). However, the sample was small (64 monozygotic (MZ) and 32 dizygotic (DZ) twin pairs), and the fraternal twin correlations were largely non-significant and showed large fluctuations across the learning trials. Another study (Hambrick and Tucker-Drob 2015) reported that the importance of genetic factors for musical achievement increased from 0 to 40% with practice. However, the study analysed group differences between practicers and non-practicers and did not assess practice hours as a continuous moderator. Most importantly, the study did not include an objective measure of expert performance, but focused on self-perceived music accomplishment, i.e., self-reported participation in music contests and public performances. Of the 269 individuals who reported a musical accomplishment, 50 participants reported to never have practiced. The authors note that it is puzzling how individuals could have accomplished any of the assessed (quite considerable) musical achievements without ever having practiced an instrument (Hambrick and Tucker-Drob 2015), which merits validation of the findings and underscores the importance of evaluating the DP theory using objective measures of expert performance (Ericsson and Lehmann 1996). 

Finally, it can be noted that there is evidence from several studies that cognitive ability influences both expert performance and the effects of practice on expertise. For instance, Meinz and Hambrick (2010) demonstrated that working memory capacity predicts sight-reading performance in pianists even when practice effects were accounted for. Similarly, Mosing and coworkers found that practice and intelligence, as well as a positive practice × intelligence interaction, have effects on both achievement and expert performance in the musical domain (Mosing et al. 2019). Recently, Wesseldijk and coworkers found similar interaction effects on musical expertise between polygenic scores for cognitive performance and practice, suggesting that the efficacy of long-term practice is moderated by genes involved in general cognitive ability (Wesseldijk et al. 2024).

In summary, a body of behaviour genetic studies have shown, contrary to the predictions of the DP theory, that genetic factors impact the acquisition of expertise, with some individuals being more talented or learning specific skills faster than others. The findings also quite strongly suggest that partly the same genes underlie practice and expertise in specific domains. Given these findings, Ullén et al. (2016) proposed an alternative framework for expertise studies which can better accommodate past research findings: the Multifactorial Gene–Environment Interaction Model (MGIM; see Supplementary Figure S1). This framework emphasises that a wide range of psychological and physical variables (depending on the domain) are relevant to expert performance. Importantly, the MGIM considers genetic factors as well as environmental (non-genetic) influences and their interaction as important for not only expert performance, but also for DP and other expertise-relevant traits (Ullén et al. 2016). While the MGIM predicts that genetic influences remain important on all levels of expertise (regardless of practice levels), one central prediction of the DP theory is that the heritability of expertise decreases with practice hours. This prediction of the DP theory has so far not been tested using measures of actual musical expertise and accumulated practice hours in a large sample.

Using music as a model, here, we test this hypothesis using a large sample of twins (N = 6471), a continuous practice measure (lifetime amount of music practice), and an objective skill score based on performance on a auditory music discrimination test—the Swedish Musical Discrimination Test (SMDT) (Ullén et al. 2014). A bivariate Cholesky decomposition (Purcell 2002) was fitted with practice as a moderator. This approach allowed us to determine to what extent genetic and environmental influences on individual differences in musical expertise are influenced by practice while taking into account gene–environment correlation between musical expertise and practice. 

## 2. Materials and Methods

Data for this study were collected in 2012 and 2013 as part of a web survey administered to an adult cohort of twins registered at the Swedish Twin Registry (Lichtenstein et al. 2002; Lichtenstein et al. 2006). The web survey was designed to collect extensive data on both music-related variables and general psychological traits. In total, 11,543 twins aged between 27 and 54 participated in the survey, but the sample for the current study is lower due to missing data (see Section 3). Zygosity was determined based on a questionnaire about intra-pair resemblance. In the Swedish Twin Registry, agreement on zygosity determination based on the intra-pair resemblance questionnaire and DNA genotyping is more than 98% (Lichtenstein et al. 2002, 2006). For further information on the data collection procedure and the web survey, see Mosing et al. (2014a).

*Musical expertise* was operationalised as music auditory discrimination and measured with the Swedish Musical Discrimination Test (SMDT), which includes three subscales measuring pitch, melody, and rhythm discrimination (Ullén et al. 2014). During the pitch subtest (27 trials) participants are presented with two successive tones that differ in pitch. Participants are asked to indicate whether the second tone is lower or higher than the first. During the melody subtest (18 trials), participants are presented with two isochronous sequences of four to nine tones. The pitch of one randomly selected tone is altered in the second stimulus in such a way that the melodic contour of the sequence is not changed. Participants are asked to indicate which tone in the second sequence differs from the first. In the rhythm subtest (18 trials), participants are presented with two rhythmical sequences of five to seven tones (all with the same pitch) that are the same or different from each other. Participants are asked to indicate whether the two rhythmical sequences are the same or different. Internal consistencies and split-half reliabilities of the three subscales are generally high (ranging between 0.79 and 0.89). For a detailed description and psychometric validation of the SMDT, see Ullén et al. (2014).

We summed the number of correct trials per subtest, resulting in a score for rhythm, melody, and pitch discrimination. A measure of overall musical expertise was obtained by taking the mean of the standardised subscale scores (if participants had valid scores on all subtests). Subsequently, age and sex effects were regressed out, and the residual scores were winsorised at three standard deviations from the mean and standardised separately by sex.

*Lifetime amount of music practice* was determined based on retrospective self-report questions. First, participants were asked whether they had ever played an instrument or had actively sung. Those who responded positively were then asked about average weekly practicing intensity (in 10 categories ranging from 0 to over 6–9 and to more than 40 h) during four age intervals (age ranges of 0–5 years, 6–11 years, and 12–17 years and from 18 years to the date of measurement). Taking into account the start age (and end age, when applicable), we calculated an estimate of cumulative lifetime amount of music practice by multiplying the weeks/years practiced in each age category × intensity category and then summing up the sums of the four age intervals. Participants that indicated that they had never played an instrument or actively sung were given a music practice score of zero. Subsequently, age and sex effects were regressed out, and the residuals were winsorised at three standard deviations from the mean and standardised separately by sex.

We fitted biometrical genetic models to determine the extent to which individual differences in musical expertise were due to genetic and environmental influences, and to test whether these influences were moderated by lifetime music practice. By applying the classical twin design, it is possible to decompose the variance in musical expertise into additive genetic (A), shared environmental (C), and residual (E) influences (Neale and Cardon 1992). Additive genetic variance is the influence of the summed allelic effects. Shared environmental variance results from environmental influences shared within pairs growing up together which make them more similar to each other, such as the family environment and neighbourhood they grow up in. Residual variance results from influences not shared by twin pairs, including unique environmental influences not shared between twins, stochastic biological effects, as well as measurement errors. 

Identical (monozygotic, MZ) twins share all of their genes, whereas non-identical (dizygotic, DZ) twins, on average, share 50% of their segregating genes. Therefore, if A was the only source of variance in a trait, we would expect a twin pair correlation of 1 for MZ pairs, while for DZ pairs, the twin correlation would be 0.5. If C was the only source of variance in a trait, we would expect a twin pair correlation of 1 for both MZ and DZ twin pairs. Finally, if all variances were due to E, we would expect a twin pair correlation of 0 for both MZ and DZ twin pairs. Hence, A, C, and E influences predict different patterns of MZ versus DZ twin pair correlations, and we used structural equation modelling to determine which combination best matched our observed data. 

We conducted biometrical analyses using maximum likelihood procedures in the statistical package Mx (Neale et al. 2006a). In maximum-likelihood modelling, the goodness-of-fit of a model to the observed data is distributed as chi-square (χ^2^). To test whether dropping model parameters or constraining parameters to be equal significantly worsened the model fit, we tested the change in the chi-square value (Δχ^2^) against the change in degrees of freedom (Δdf). 

**Figure 1:**
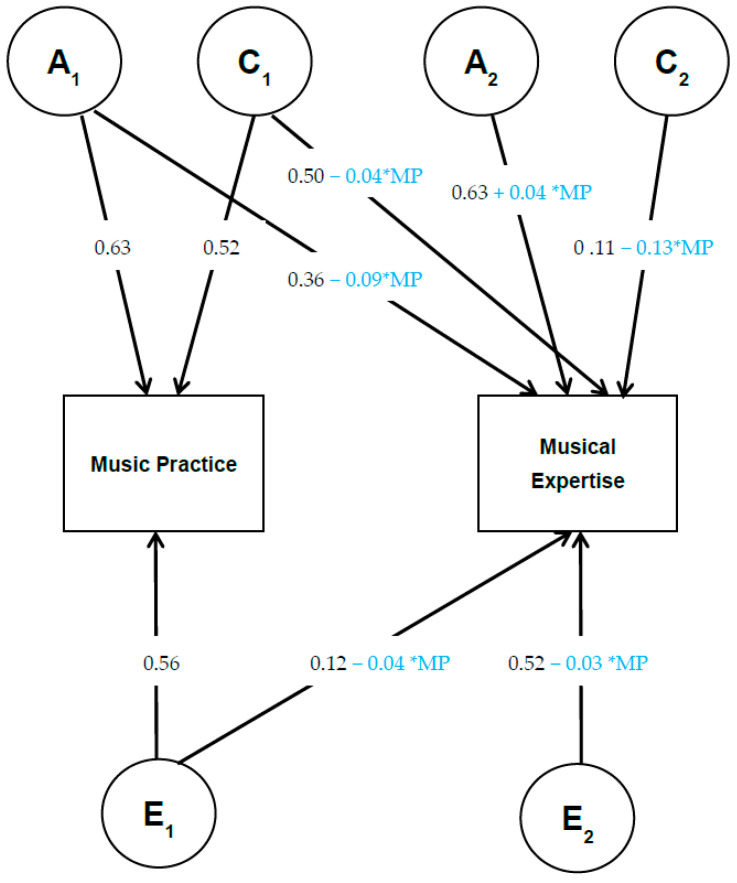
The bivariate moderator model with practice hours as the moderator variable. The boxes represent the observed variables, and the circles represent the latent variables that influence the observed variables. A, C, and E denote the latent genetic, shared environmental, and residual influences, respectively. A_1_ is a genetic factor that influences both traits (Music practice and musical expertise) accounting for shared genetic (co-)variation between the traits (genetic overlap or genetic pleiotropy), while A_2_ only influences musical expertise. The same structure applies for the C and E factors. The blue numbers represent the moderator effect of the lifetime amount of music practice (MP) on the path estimate.

To test whether there is evidence of a gene–environment (G × E) interaction, we estimated that the A, C, and E variance components are conditional upon lifetime music practice. If there is no G × E interaction, the estimates for the genetic effects should not differ between individuals with more versus less music practice. On the other hand, there is evidence for a G × E interaction when the estimates of the proportion of genetic variance are dependent on the level of lifetime music practice. There are several approaches for examining G × E interaction in twin designs (Purcell and Sham 2002; Rathouz et al. 2008; van der Sluis et al. 2012). Here, we used the bivariate model as described by Purcell and Sham (2002). This model takes into account the gene–environment correlation (rGE) between the moderator and the outcome variable, i.e., the extent to which the same versus different genetic and environmental factors contribute to the moderator and the outcome phenotype (see Figure 1). When the genetic influences that account for variance in practice also explain the variance in musical expertise, this indicates that there is a gene–environment correlation. A previous study on the same twin cohort studied here showed that the association between music practice and musical expertise can be partly explained by overlapping genetic influences (Mosing et al. 2014a), providing evidence for the presence of a gene–environment correlation. Therefore, it is important to account for rGE because if it is not explicitly modelled, rGE may incorrectly be reflected as G × E (Purcell and Sham 2002). 

As shown in Figure 1, the bivariate model includes genetic and environmental paths that are shared between lifetime music practice and musical expertise (crosspaths) as well as genetic and environmental influences that are unique to musical expertise. As such, we estimate the extent to which overlapping and unique A, C, and E factors influence musical expertise. To incorporate moderation influences, both the overlapping and unique A, C, and E influences on musical expertise are moderated by practice hours. Accordingly, with the bivariate moderator model, it is possible to distinguish moderation on the crosspaths from moderation on the unique paths. For instance, the genetic influences on musical expertise include those overlapping with practice hours (a_21_) and those specific to musical expertise (a_22_). Both of these genetic parameters are moderated by practice hours such that the overall genetic influences on musical expertise are (a_21_ + β_1_ × lifetime music practice) and (a_22_ + β_2_ × lifetime music practice). Both β_1_ and β_2_ represent gene–environment interaction, but the former represents G × E interaction for A influences on musical expertise that are shared with lifetime music practice, while the latter represents G × E interaction for A influences unique to musical expertise. The presence of moderation on the A, C, and E paths was tested by dropping the moderator effect to zero and comparing the model fit. We also compared the fit of the moderator models with a model which included no moderation. As an additional sensitivity analysis, to make sure that the results are not driven by differences between those who play an instrument and those who do not (i.e., zero practice versus practice), all analyses were repeated with a restricted sample including only twin pairs where both played an instrument (see Supplementary Table S2 and Figure S2).

## 3. Results

After excluding twins with unknown zygosity (N = 368) and with missing data on the musical expertise test and/or lifetime music practice variable (N = 4695 and N = 754, respectively), the final study sample contained 6471 twins (58.1% females). This included 1302 complete twin pairs (240 MZ male, 442 MZ female, 133 DZ male, 202 DZ female, and 285 DZ opposite-sex (DZOS) pairs) and 3867 single twins whose co-twin did not participate. The mean age was 40.7 years (SD = 7.8). The relatively high number of drop-outs is due to the fact that the SMDT was administered close to the end of the online test battery, which took 50 to 120 min to complete. Sample descriptives of the music discrimination subtests and the hours of music practice can be found in Table 1. Overall, 61.4% of males and 79.1% of females indicated they had played an instrument or sung actively. Females on average reported more practice hours than males (Cohen’s d = 0.10, *p* < 0.001), but when only considering participants that had played an instrument or sung actively, males on average reported more practice hours than females. 

Males scored somewhat higher on overall musical expertise than females (Cohen’s d = 0.13, *p* < 0.001). The phenotypic correlation between lifetime practice and aptitude was 0.36 (*p* < 0.0001), and individuals who had played an instrument or sung actively scored significantly higher on musical expertise than individuals who had not (Cohen’s d = 0.77 for males and 0.78 for females, both *p* < 0.001). As expected, older participants reported a higher lifetime amount of music practice (β = 0.09 for males and β = 0.17 for females, both *p* < 0.001). However, they scored lower on musical expertise (β = −0.13 and *p* < 0.001 for males and β = −0.06 and *p* = 0.001 for females). Note that the effects of sex and age on the means were accounted for in the genetic analyses.

Prior to fitting the moderator model, we tested whether the means and variances of the two variables were comparable across zygosities (α = 0.05). For musical expertise, no significant differences in the means or variances were found between the zygosity groups. For lifetime music practice, we found no significant mean differences between zygosity groups, but the variance was larger in the DZ females than MZ females (Δχ^2^(1) = 10.93; *p* < 0.001) as well as in the DZ males compared to MZ males (Δχ^2^(1) = 10.73; *p* = 0.001). 

Table 2 shows the twin pair correlations for both variables for each zygosity group. The opposite-sex twin pair correlations for musical expertise were significantly lower than the DZ same-sex twin pair correlations (Δχ^2^(1) = 6.65; *p* = 0.01). For lifetime amount of music practice, the opposite-sex twin pair correlations were nominally lower than the DZ same-sex twin pair correlations, but this difference was not significant (Δχ^2^(1) = 3.28; *p* = 0.07). However, simultaneously equating the DZ male, female, and opposite-sex twin pair correlations resulted in a significant deterioration of model fit (Δχ^2^(2) = 6.72; *p* = 0.03). A lower opposite-sex DZ twin pair correlation than same-sex twin pair correlation indicates qualitative sex differences in the sources of variation, i.e., differences in the sources of genetic and/or shared environmental variation between males and females. It is possible to model qualitative sex differences by allowing the genetic component to have a correlation of less than 0.5 between DZ opposite-sex twins or the shared environmental component to have a correlation of less than 1 between DZ opposite-sex twins. However, DZ opposite-sex twins do not provide sufficient information to estimate a reduced correlation for both A and C components. Furthermore, the extension of this approach to multivariate Cholesky decompositions is not straightforward and results in identification issues (see Neale et al. 2006b). For that reason, we did not include opposite-sex twins in subsequent genetic modelling. 

We then fitted univariate models to estimate the genetic (A), shared environmental (C), and residual (E) variance components. We started with a common-effects sex limitation model, which allows for quantitative sex differences in the variance components. We tested for quantitative sex differences by constraining the variance estimates to be equal across sexes. For both variables, male and female path estimates could be equated without significant deterioration of the model fit (Δχ^2^(2) = 1.44 and *p* = 0.49 for musical expertise and Δχ^2^(2) = 5.72 and *p* = 0.06 for practice). Therefore, variance components were estimated for both sexes and combined in subsequent modelling. The univariate Cholesky decomposition (which does not take into account the gene–environment correlation and interaction) indicated that individual differences in musical expertise are 50% (95% confidence intervals (CIs): 35–67%) due to genetic influences and 22% (95% CIs: 5–36%) and 28% (25–32%) due to C and E influences, respectively.

Subsequently, we fitted a bivariate moderator model to test whether the A, C, and E influences on musical expertise were moderated by lifetime music practice while taking into account the gene–environment correlation between the two variables. Estimates of the genetic and environmental pathways along with the moderator effects on these paths can be found in Figure 1. The model fitting results show that the moderator effects on all six pathways cannot be dropped simultaneously from the model without significant deterioration of model fit, although none of the individual moderator effects are significant (Table 3). Also, the moderator effects on all three crosspaths could not be dropped from the model simultaneously without deterioration of model fit (*p* < 0.001), demonstrating a significant influence of lifetime music practice on the covariation between musical expertise and music practice.

**Figure 2:**
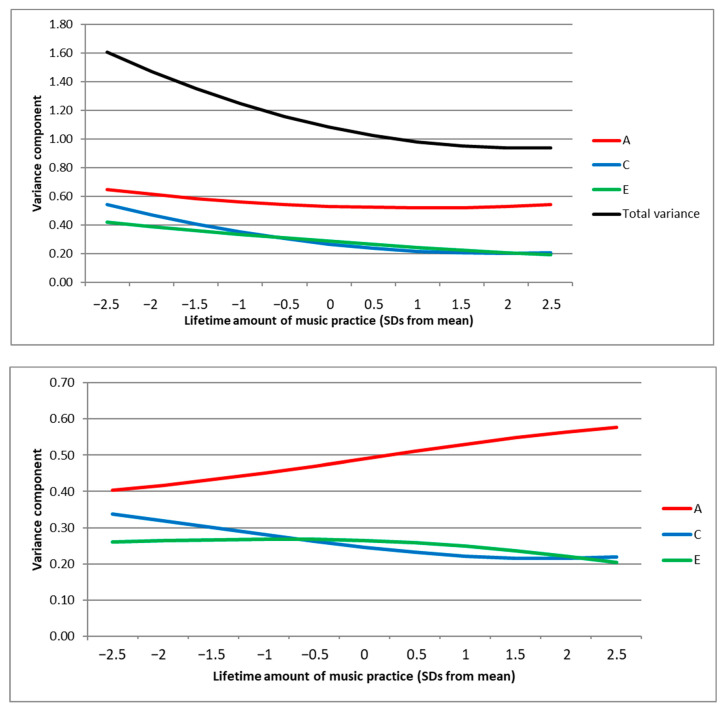
(**Top Panel**): Estimates of total variance and genetic influence (A), shared environmental influence (C), and residual influence (E) components of variation in musical expertise depending on level of lifetime amount of music practice (ranging from −2.5 to +2.5 SDs from mean). (**Bottom Panel**): Relative contribution of A, C, and E influences on musical expertise depending on level of lifetime amount of music practice.

Simultaneously dropping the moderator effects on the two residual latent factors E1 and E2 led to a significantly worse model fit (*p* = 0.02), showing that with more practice, the overall residual influences on variation in musical expertise significantly decreased. In contrast, simultaneously dropping the moderator effects on A1 and A2 or C1 and C2 did not lead to a significantly worse model fit. Figure 2 (top panel) illustrates that the overall variance in musical expertise diminishes with more practice hours, as do the C and E variance components (although the decrease is not significant for C). The A influences on musical expertise stay relatively stable in absolute terms, with decreasing genetic influences that are shared with lifetime music practice and increasing unique genetic influences with more practice hours (see Figure 1). The bottom panel of Figure 2 illustrates that the relative contribution of A on the variation in musical expertise therefore tends to increase at higher levels of lifetime music practice, whereas the relative contributions of C and E slightly decrease. Sensitivity analyses with a restricted sample including only twins that had played an instrument showed the same pattern of results, although the moderation effects on the latent E1 and E2 variance components were also independently significant in this case (see Supplementary Table S2 and Figure S2).

## 4. Discussion

In this study, we used a large twin sample to explore the potential moderation effects of musical practice on the genetic and environmental influences on musical expertise (operationalised as music auditory discrimination). Specifically, we tested whether genetic influences on musical expertise decrease with practice, as predicted by the DP theory (see Ericsson 2007). The results do not support this hypothesis. In absolute terms, while the overall variance significantly decreased as a function of practice due to a decrease in residual variance, the genetic influences on musical expertise remained relatively constant, resulting in an increase in the heritability (i.e., the proportion of genetic variance) of musical expertise with practice. 

In line with past research (Coon and Carey 1989; Drayna et al. 2001; Ullén et al. 2014; Vinkhuyzen et al. 2009), our results show that over the whole sample, approximately 50% of individual differences in musical expertise can be explained by genetic differences between individuals, whereas shared environmental and residual influences each explain about 25%. As we have already shown previously (see Mosing et al. 2014a), the genetic influences on musical expertise partly overlap with those underlying lifetime music practice, indicating that partly the same genes influence musical ability and the willingness to practice. Even more important, our bivariate twin model showed that music practice significantly moderates the variance component estimates of musical expertise. The overall variation in musical expertise decreased with more practice hours, which was due to a decrease in shared environmental and residual variance components (although the decrease in shared environmental variance was not significant). With more practice hours, environmental influences thus tend to play a smaller role of variation in musical expertise. The decrease in overall variance implies that differences between individuals in musical expertise are smaller in participants with a higher level of practice. In contrast, the absolute genetic variation remained relatively stable as a function of practice, i.e., the overall genetic influence on expertise was unaffected by the number of practice hours. Importantly, the relative contribution of genetic influences thus tends to become larger with more practice hours, i.e., the heritability of musical expertise tends to increase with more practice. The results of the sensitivity analyses in only twins who play an instrument further support these findings.

As such, our findings are inconsistent with the predictions made by the DP theory (Ericsson 2014a; Ericsson et al. 1993, 2005; Ericsson and Ward 2007), which argues that genetic factors become less important with increasing practice, and that the effects of genetic factors on performance are eventually eliminated. To reiterate, our results indicate that the opposite is true: in relative terms, genetic factors become more important with increasing practice. 

The present findings demonstrate the importance of genetic predispositions in (musical) expertise, and such genetic predispositions for expertise in a given field remain important even after many hours of practice. This, in combination with other recent findings (Ullén et al. 2016; Wesseldijk et al. 2023), strongly suggests that although practice certainly plays an important role in becoming an expert musician, practice alone will not be sufficient. This is in line with studies suggesting that the amount of practice needed to reach a specific expertise level will differ between individuals (Mosing et al. 2019). This may likely also extend to other fields of expertise, as already suggested by some studies (e.g., Fox et al. 1996; Vinkhuyzen et al. 2009), including (other) arts as well as sports and other domains involving physical or mental activities. 

An interesting finding is that the overall variance in musical expertise decreased with practice. Since earlier analyses in the same cohort have found associations between practice and expertise likely not to reflect causal effects of practice on expertise (Mosing et al. 2014a), one plausible explanation is that this phenomenon reflects attrition, i.e., that individuals with a lower level of aptitude for music tend to quit practicing. Another possibility is that practice has a larger effect when skill levels are low, resulting in reduced residual variance and therefore in reduced overall variance. Furthermore, initial genetic differences may be amplified during training (or maintained) because of the gene–environment correlation, where individuals are exposed to environments that are correlated with their genetic propensities. For example, children that are good at singing may be more likely to join a choir than less talented children.

The Multifactorial Gene–Environment Interaction Model (MGIM) emphasises that a wide range variables, including genetic factors as well as environmental (non-genetic) influences and their interaction, are relevant for expert performance, DP, and other expertise-relevant variables (Ullén et al. 2016). Importantly, it poses that genetic influences remain important on all levels of expertise. As such, the present findings provide further support for the MGIM, emphasising the importance of genetic influences on expertise and suggesting gene–environment (G × E) interaction and correlation between practice and musical expertise.

Although our continuous measures of musical expertise and practice represent an important step forward compared to earlier studies, some limitations should be considered. First, we focused on one aspect of musical expertise, i.e., the ability to perceptually discriminate musically relevant sounds rather than the overall ability to play an instrument. However, the ability to discriminate melodies, rhythms, and pitches is obviously fundamental for musical expertise, and musicians systematically outperform non-musicians in auditory discrimination tasks (Schellenberg and Weiss 2013). Furthermore, it should be noted that sensory and motor processes are highly integrated in performance; motor control and learning thus critically depends on the efficient processing of sensory feedback (see e.g., Shadmehr et al. 2010). In supplementary analyses, we show that our measure of auditory discrimination significantly predicts 19% of the variance in real-life musical achievement (music scale of the Creative Achievement Questionnaire; Carson et al. 2005) and 13% of the variance in musical motor skills (temporal precision in rhythmic motor tasks; Madison 2001) and that associations hold even when adjusting for IQ (Formann and Piswanger 1979; Mosing et al. 2016). See the Supplementary Information and Supplementary Table S1 for more details on the validation analyses. 

Lifetime music practice measures were derived based on self-reports of average weekly practice intensity in four age intervals. A retrospective estimation of practice is a common method used in many studies on expertise (see e.g., Duffy et al. 2004; Ericsson et al. 1993; Tuffiash et al. 2007), but it should be acknowledged that such measures of practice may be subject to recall inaccuracy. It should also be noted that estimates of lifetime practice were positively skewed with many subjects having no or little practice. As shown in previous papers using these data (Mosing et al. 2015; Mosing et al. 2014b), transformation of the variable or the exclusion of individuals with no practice at all enhanced normality but did not result in a normal distribution. The twin pair correlations and variance component estimates of the untransformed and transformed musical expertise measure were very similar (Mosing et al. 2014a; Mosing et al. 2015). In general, the assumption of normality is not easily violated if the sample is large (based on the central limit theorem, see Field 2009), and maximum likelihood methods show robustness to violations of the assumption of multivariate normality (Kaplan 1990). As the transformed and untransformed results were very similar and we have a large sample, we used the untransformed data for our analyses.

Finally, we were unable to drop the moderator effect from the bivariate model without significant deterioration of model fit, indicating that practice significantly influenced the variance component estimates of musical expertise. However, the effects of the moderator on the individual pathways were not significant, so we cannot make specific claims about the effect of practice on single paths. Also, the moderator effects on the (combined) E influences on musical expertise were significant, but the moderator effects on the A and C influences were not. The power to detect moderation on the E variance components is much greater than the power to detect the moderation of A or C variance components (van der Sluis et al. 2012). 

To conclude, our study shows that music practice significantly moderates the architecture of musical expertise. The overall variation in musical expertise decreased with more practice hours. While the shared environmental and residual variance components decreased with more practice, the genetic variation remained relatively stable, resulting in an increase in heritability (i.e., the proportion of genetic variance) with more practice. These findings are inconsistent with predictions made by the DP theory, as we showed that genetic factors play an important role in musical expertise and remain important even after many practice hours. At a general level, these results are in line with newer multifactorial models of expertise in which practice still plays an essential role in becoming an expert, but it is not assumed to be sufficient to reach the highest level of skill. Future studies are needed to test whether the same findings hold in other domains of expertise. 

## Figures and Tables

**Table 1:** Descriptive statistics.

	Males			Females		
	N	Range	M (SD)	N	Range	M (SD)
Musical expertise ^1^						
- Pitch	2712	1–27	18.7 (5.1)	3759	1–27	17.8 (4.6)
- Melody	2712	0–18	6.8 (3.0)	3759	0–17	6.6 (2.8)
- Rhythm	2712	4–18	15.4 (2.2)	3759	5–18	15.3 (2.2)
Lifetime music practice ^2^	2712	0–23,920	2380 (3699)	3759	0–22,776	2734 (3651)
Lifetime music practice (in participants that had played an instrument or had actively sung) ^3^	1665	52–23,920	3876 (4061)	2973	52–22,776	3457 (3789)

M = mean; SD = standard deviation. ^1^ The musical expertise variable is the mean of the standardised scale scores of the rhythm, melody, and pitch music discrimination tests; descriptives are provided for the sub-tests. ^2^ Note that these are estimates based on range categories of weekly practice hours. ^3^ The sample used in the supplementary analyses with only playing twins.

**Table 2:** Twin pair correlations (and 95% confidence intervals) for musical expertise and lifetime amount of music practice as estimated in Mx.

Zygosity	Musical Expertise	Lifetime Music Practice
MZ M	0.72 (0.66–0.77)	0.68 (0.61–0.73)
MZ F	0.72 (0.68–0.76)	0.64 (0.59–0.69)
DZ M	0.53 (0.40–0.62)	0.61 (0.50–0.69)
DZ F	0.43 (0.32–0.53)	0.44 (0.32–0.54)
DZ OS	0.31 (0.21–0.40)	0.40 (0.30–0.49)

F = female, M = male, MZ = monozygotic, DZ = dizygotic, OS = opposite sex.

**Table 3:** The genetic modelling results showing the changes in model fit (Δχ^2^) and degrees of freedom (Δdf) when the specified parameters are dropped from the full model.

Model	Δχ^2^	Δdf	*p*-Value
Full bivariate moderator model			
Drop moderator effect all paths	45.13	6	<0.001
Drop moderator effect all crosspaths	34.83	3	<0.001
Drop moderator effect all unique paths	3.87	3	0.28
Drop moderator on A (A1 and A2)	1.73	2	0.42
Drop moderator on C (C1 and C2)	0.94	2	0.62
Drop moderator on E (E1 and E2)	7.54	2	0.02
Drop moderator on crosspath A1	1.25	1	0.26
Drop moderator on crosspath C1	0.19	1	0.66
Drop moderator on crosspath E1	2.84	1	0.09
Drop moderator on unique path A2	0.35	1	0.55
Drop moderator on unique path C2	0.92	1	0.34
Drop moderator on unique path E2	3.66	1	0.06

A1 and A2 refer to the first and second latent genetic components, C1 and C2 refer to the first and second latent shared environmental components, and E1 and E2 refer to the first and second latent residual components (see Figure 1).

## Data Availability

The datasets generated during the current study cannot be made publicly available as registry data were used. However, researchers are able to apply online at the Swedish Twin Registry to access the twin data.

## Supplementary Materials

The following supporting information can be downloaded at https://www.mdpi.com/article/10.3390/jintelligence12090087/s1; Supplementary information and analyses: The validity of the musical auditory discrimination test as a measure of musical expertise; Table S1: Association of musical auditory discrimination with musical creative achievement and motor timing; Table S2: The genetic modelling results in the reduced sample of only playing twins showing the change in model fit (Δχ^2^) and degrees of freedom (Δdf) when the specified parameters are dropped from the full model; Figure S1: The Multifactorial Gene–Environment Interaction Model (MGIM) (Ullén et al. 2015); Figure S2: The modelling results in the reduced sample with only playing twins.

## References

[B1-jintelligence-12-00087] Branigan Amalia R., McCallum Kenneth J., Freese Jeremy (2013). Variation in the Heritability of Educational Attainment: An International Meta-Analysis. Social Forces.

[B2-jintelligence-12-00087] Carson Shelley, Peterson Jordan B., Higgins Daniel M. (2005). Reliability, validity, and factor structure of the creative achievement questionnaire. Creative Research Journal.

[B3-jintelligence-12-00087] Cesarini David, Visscher Peter M. (2017). Genetics and educational attainment. NPJ Science of Learning.

[B4-jintelligence-12-00087] Coon Hilary, Carey Gregory (1989). Genetic and environmental determinants of musical ability in twins. Behavior Genetics.

[B5-jintelligence-12-00087] Detterman Douglas K., Ruthsatz Joanne M. (1999). Toward a more comprehensive theory of exceptional abilities. Journal for the Education of the Gifted.

[B6-jintelligence-12-00087] Drayna Dennis, Manichaikul Ani, Lange Marloes de, Snieder Harold, Spector Tim (2001). Genetic correlates of musical pitch recognition in humans. Science.

[B7-jintelligence-12-00087] Duffy Linda J., Baluch Bahman, Ericsson Karl Anders (2004). Dart performance as a function of facets of practice amongst professional and amateur men ana women players. International Journal of Sport Psychology.

[B8-jintelligence-12-00087] Ericsson Karl Anders (2006). The Influence of Experience and Deliberate Practice on the Development of Superior Expert Performance.

[B9-jintelligence-12-00087] Ericsson Karl Anders (2007). Deliberate practice and the modifiability of body and mind: Toward a science of the structure and acquisition of expert and elite performance. International Journal of Sport Psychology.

[B10-jintelligence-12-00087] Ericsson Karl Anders (2014a). Expertise. Current Biology.

[B11-jintelligence-12-00087] Ericsson Karl Anders (2014b). Why expert performance is special and cannot be extrapolated from studies of performance in the general population: A response to criticisms. Intelligence.

[B12-jintelligence-12-00087] Ericsson Karl Anders, Lehmann Andreas C. (1996). Expert and exceptional performance: Evidence of maximal adaptation to task constraints. Annual Review of Psychology.

[B13-jintelligence-12-00087] Ericsson Karl Anders, Smith Jacqui A. (1991). Toward a General Theory of Expertise: Prospects and Limits.

[B14-jintelligence-12-00087] Ericsson Karl Anders, Ward Paul (2007). Capturing the naturally occurring superior performance of experts in the laboratory toward a science of expert and exceptional performance. Current Directions in Psychological Science.

[B15-jintelligence-12-00087] Ericsson Karl Anders, Pool Robert (2016). Peak: Secrets from the New Science of Expertise.

[B16-jintelligence-12-00087] Ericsson Karl Anders, Nandagopal Kiruthiga, Roring Roy W. (2005). Giftedness viewed from the expert performance perspective. Journal for the Education of the Gifted.

[B17-jintelligence-12-00087] Ericsson Karl Anders, Krampe Ralph T., Tesch-Römer Clemens (1993). The role of deliberate practice in the acquisition of expert performance. Psychological Review.

[B18-jintelligence-12-00087] Feltovich Paul J., Prietula Michael J., Ericsson Karl Anders (2006). Studies of Expertise from Psychological Perspectives.

[B19-jintelligence-12-00087] Field Andrew (2009). Exploring assumptions. Discovering Statistics Using SPSS.

[B20-jintelligence-12-00087] Formann Anton K., Piswanger Karl (1979). Wiener Matrizen Test [Vienna Matrices Test]: Ein Rasch-Skalierter Sprachfreier Intelligenztest.

[B21-jintelligence-12-00087] Fox Paul W., Hershberger Scott L., Bouchard Thomas J. J. (1996). Genetic and environmental contributions to the acquisition of a motor skill. Nature.

[B22-jintelligence-12-00087] Hambrick David Zach, Tucker-Drob Elliot M. (2015). The genetics of music accomplishment: Evidence for gene-environment correlation and interaction. Psychonomic Bulletin & Review.

[B23-jintelligence-12-00087] Hambrick David Zach, Macnamara Brooke N., Campitelli Guillermo, Ullén Fredrik, Mosing Miriam A. (2016). Beyond Born versus Made: A New Look at Expertise. Psychology of Learning and Motivation.

[B24-jintelligence-12-00087] Howe Michael J., Davidson Jane W., Sloboda John A. (1998). Innate talents: Reality or myth?. Behavioral and Brain Sciences.

[B25-jintelligence-12-00087] Kaplan David (1990). Evaluating and modifying covariance structure models: A review and recommendation. Multivariate Behavioral Research.

[B26-jintelligence-12-00087] Lichtenstein Paul, Sullivan Patrick F., Cnattingius Sven, Gatz Margaret, Johansson Sofie, Carlström Eva, Björk Camilla, Svartengren Magnus, Wolk Alicja, Klareskog Lars (2006). The Swedish Twin Registry in the third millennium: An update. Twin Research and Human Genetics.

[B27-jintelligence-12-00087] Lichtenstein Paul, Faire Ulf De, Floderus Birgitta, Svartengren Magnus, Svedberg Pia, Pedersen Nancy L. (2002). The Swedish Twin Registry: A unique resource for clinical, epidemiological and genetic studies. Journal of Internal Medicine.

[B28-jintelligence-12-00087] Madison Guy (2001). Variability in isochronous tapping: Higher-order dependencies as a function of inter tap interval. Journal of Experimental Psychology: Human Perception and Performance.

[B29-jintelligence-12-00087] Meinz Elizabeth J., Hambrick David Z. (2010). Deliberate practice is necessary but not sufficient to explain individual differences in piano sight-reading skill: The role of working memory capacity. Psychological Science.

[B30-jintelligence-12-00087] Mosing Miriam A., Hambrick David Z., Ullén Fredrik (2019). Predicting musical aptitude and achievement: Practice, teaching, and intelligence. Journal of Expertise.

[B31-jintelligence-12-00087] Mosing Miriam A., Madison Guy, Pedersen Nancy L., Ullén Fredrik (2015). Investigating cognitive transfer within the framework of music practice: Genetic pleiotropy rather than causality. Developmental Science.

[B32-jintelligence-12-00087] Mosing Miriam A., Madison Guy, Pedersen Nancy L., Kuja-Halkola Ralph, Ullén Fredrik (2014a). Practice does not make perfect: No causal effect of musical practice on musical ability. Psychological Science.

[B33-jintelligence-12-00087] Mosing Miriam A., Verweij Karin J. H., Madison Guy, Ullén Fredrik (2016). The genetic architecture of correlations between perceptual timing, motor timing, and intelligence. Intelligence.

[B34-jintelligence-12-00087] Mosing Miriam A., Pedersen Nancy L., Madison Guy, Ullén Fredrik (2014b). Genetic pleiotropy explains associations between musical auditory discrimination and intelligence. PLoS ONE.

[B35-jintelligence-12-00087] Neale Michael C., Cardon Lon R. (1992). Methodology for Genetic Studies of Twins and Families.

[B36-jintelligence-12-00087] Neale Michael C., Røysamb Espen, Jacobson Kristen (2006a). Multivariate genetic analysis of sex limitation and G × E interaction. Twin Research and Human Genetics.

[B37-jintelligence-12-00087] Neale Michael C., Boker Steven M., Xie Gary, Maes Hermine H. (2006b). Mx: Statistical Modeling.

[B38-jintelligence-12-00087] Plomin Robert, Shakeshaft Nicholas G., McMillan Andrew, Trzaskowski Maciej (2014). Nature, Nurture, and Expertise. Intelligence.

[B39-jintelligence-12-00087] Purcell Shaun (2002). Variance components models for gene-environment interaction in twin analysis. Twin Research.

[B40-jintelligence-12-00087] Purcell Shaun, Sham Pak (2002). Variance components models for gene-environment interaction in quantitative trait locus linkage analysis. Twin Research.

[B41-jintelligence-12-00087] Rathouz Paul J., Hulle Carol A. Van, Rodgers Joseph Lee, Waldman Irwin D., Lahey Benjamin B. (2008). Specification, testing, and interpretation of gene-by-measured-environment interaction models in the presence of gene-environment correlation. Behavior Genetics.

[B42-jintelligence-12-00087] Ruthsatz Joanne, Detterman Douglas, Griscom William S., Cirullo Britney A. (2008). Becoming an expert in the musical domain: It takes more than just practice. Intelligence.

[B43-jintelligence-12-00087] Schellenberg Glenn, Weiss Michael W. (2013). Music and Cognitive Abilities.

[B44-jintelligence-12-00087] Shadmehr Reza, Smith Maurice A., Krakauer John W. (2010). Error correction, sensory prediction, and adaptation in motor control. Annual Review of Neuroscience.

[B45-jintelligence-12-00087] Tuffiash Michael, Roring Roy W., Ericsson Karl Anders (2007). Expert performance in SCRABBLE: Implications for the study of the structure and acquisition of complex skills. Journal of Experimental Psychology: Applied.

[B46-jintelligence-12-00087] Ullén Fredrik, Hambrick David Zach, Mosing Miriam A. (2016). Rethinking expertise: A multifactorial gene-environment interaction model of expert performance. Psychological Bulletin.

[B47-jintelligence-12-00087] Ullén Fredrik, Mosing Miriam A., Madison Guy (2015). Associations between motor timing, music practice, and intelligence studied in a large sample of twins. Annals of the New York Academy of Sciences.

[B48-jintelligence-12-00087] Ullén Fredrik, Mosing Miriam A., Holm Linus, Eriksson Helene, Madison Guy (2014). Psychometric properties and heritability of a new online test for musicality, the Swedish Musical Discrimination Test. Personality and Individual Differences.

[B49-jintelligence-12-00087] van der Sluis Sophie, Posthuma Danielle, Dolan Conor V. (2012). A note on false positives and power in G × E modelling of twin data. Behavior Genetics.

[B50-jintelligence-12-00087] Vinkhuyzen Annabelle A., Sluis van der Sophie, Posthuma Danielle, Boomsma Dorrt I. (2009). The heritability of aptitude and exceptional talent across different domains in adolescents and young adults. Behavior Genetics.

[B51-jintelligence-12-00087] Wesseldijk Laura W., Ullén Fredrik, Mosing Miriam A. (2023). Music and Genetics. Neuroscience & Biobehavioral Reviews.

[B52-jintelligence-12-00087] Wesseldijk Laura W., Mosing Miriam A., Ullén Fredrik (2024). Gene-environment interaction in expertise acquisition: Practice effects on musical expertise vary by polygenic scores for cognitive performance. Heliyon.

