# Supplementary material for: Testing the Deliberate Practice Theory: Does Practice Reduce the Heritability of Musical Expertise?
Source: J Intell. 2024 Sep 8;12(9):87. doi: 10.3390/jintelligence12090087 (PMC11433073; doi:10.3390/jintelligence12090087)
Supplement: Supplementary file 1 [file jintelligence-12-00087-s001.zip › jintelligence-3143503-supplementary.pdf]

Supplementary Material

Testing the Deliberate Practice Theory: Does Practice Reduce the Heritability of Musical Expertise?

Miriam A. Mosing, Karin J. H. Verweij, David Z. Hambrick, Nancy L. Pedersen and Fredrik Ullén

Supplementary Material

Supplementary information. Validity of Musical auditory discrimination test as a measure of musical expertise

In order to show that the musical auditory discrimination measure is a valid indicator of musical expertise, we determined its association with real life musical achievement (music scale of the Creative Achievement Questionnaire) and a motor timing task (temporal precision in rhythmic motor tasks). Analyses were also repeated while controlling for general intelligence. Measures of musical creative achievement, motor timing, and IQ were obtained as part of the same web survey in which lifetime amount of music practice and musical auditory discrimination were measured.

Music achievement was measured with an adapted version of the Creative Achievement Questionnaire (CAQ; Carson, Peterson & Higgins, 2005), a self-report inventory addressing involvement in different domains of arts and science. The music item consisted of seven statements about music achievement, ranging from 1) 'I am not engaged in music at all' via 4) 'I have played or sung, or my music has been played in public concerts in my home town, but I have not been paid for this' to 7) 'I am professionally active as a musician and have been reviewed/featured in the national or international media and/or have received an award for my musical activities'.

Motor timing was measured with the Isochronous Serial Interval Production task (ISIP, see Madison, 2001), a simple, repetitive task in which participants perform self-paced isochronous tapping movements, for instance with a finger. After a training trial with an inter onset interval (IOI) of 581ms between sounds, participants completed six experimental trials with IOIs of 524, 819, 655, 1,024, 655, and 524ms, respectively. Each ISIP trial consisted of a synchronization phase where participants tapped in synchrony with a regular, auditory metronome, followed by a continuation phase where they continued tapping self-paced without a metronome. For the analyses we used the data from the continuation phases. The final variable was created by calculating the mean coefficient of variation (SD/mean) across the six trials. More information about the ISIP tasks and data cleaning can be found elsewhere (Madison, 2001; Mosing et al., 2016).

General intelligence was measured with the Wiener Matrizen Test, a 24-item matrix test, similar to the Raven matrices task (Formann & Piswanger, 1979). Participants' IQ score was calculated as the total number of correct responses.

We used Generalized Estimating Equation (GEE) modelling in SPSS to test the association of musical auditory discrimination with music achievement and motor timing. In the GEE model, a covariance matrix is used to account for family relatedness and tests are based on robust standard error. Age and sex were included as covariates in the model. Analyses were also repeated with IQ as an additional covariate. Variance explained was calculated in regression analyses as the R2 of the model including the prediction variable (music achievement or motor timing) and covariates minus the R2 of the model including only covariates. The correlation was calculated with a partial regression, taking age, sex (and IQ) effects into account.

Analyses show that musical auditory discrimination scores significantly predict both musical creative achievement and motor timing (see Table S1 below). The musical auditory discrimination scores predict approximately 19% of the variance in real life musical achievement and 13% of the variance in musical motor skills. This indicates that musical auditory discrimination is a valid index not just of auditory discrimination but of general musical competence.

Supplementary Table S1. Association of musical auditory discrimination with musical creative achievement and motor timing.

	B	p-value	r	R^2
Creative Achievement	0.78	<0.001	0.43	0.19
Creative Achievement (controlled for IQ)	0.77	<0.001	0.41	0.16
Motor timing	-0.54	<0.001	-0.37	0.13
Motor timing (controlled for IQ)	-0.44	<0.001	-0.34	0.11

R2=variance explained, all analyses were corrected for age and sex effects

Supplementary Table S2. Genetic modelling results in the reduce sample of only playing twins showing the change in model fit (Δχ2) and degrees of freedom (Δdf) when the specified parameters are dropped from the full model.

Model	Δχ2	Δdf	p-value
Full Bivariate moderator model			
Drop moderator effect all paths	32.59	6	<0.001
Drop moderator effect all crosspaths	19.80	3	<0.001
Drop moderator effect all unique paths	6.33	3	0.10
Drop moderator on A (A1 and A2)	0.77	2	0.68
Drop moderator on C (C1 and C2)	0.50	2	0.78
Drop moderator on E (E1 and E2)	13.54	2	<0.01
Drop moderator on crosspath A1	0.54	1	0.46
Drop moderator on crosspath C1	0.12	1	0.73
Drop moderator on crosspath E1	5.21	1	<0.05
Drop moderator on unique path A2	0.43	1	0.51
Drop moderator on unique path C2	0.08	1	0.78
Drop moderator on unique path E2	6.25	1	<0.05

A1 and A2 refer to the first and second latent genetic components, C1 and C2 refer to the first and second latent shared-environmental components, E1 and E2 refer to the first and second latent residual components (see Figure 1)

Supplementary Figure S1. The Multifactorial Gene-Environment Interaction model (MGIM) of expertise (adapted from Ullén et al (2016), Psychological Bulletin.)

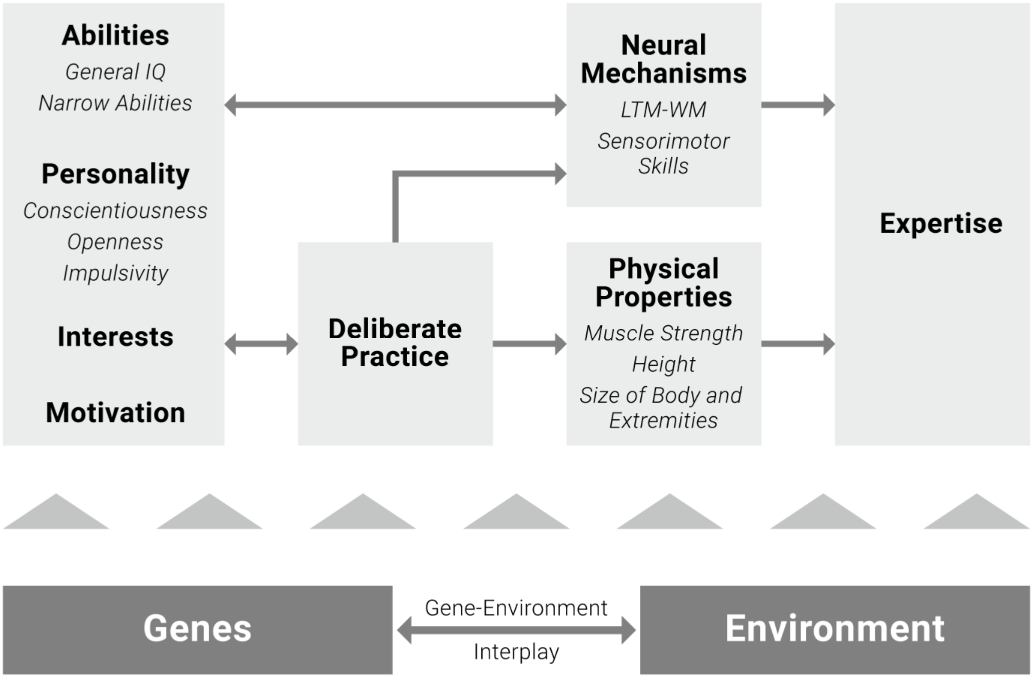

Supplementary Figure S2. Modelling results in the reduced sample with only playing twins. Top Panel: Raw estimates of total variance and genetic (A), shared environmental (C) and residual influences (E) variance components of variation in Musical Expertise as dependent on the level of lifetime amount of music practice (ranging from −2.5 to +2.5 SDs from the mean). Bottom Panel: Relative contribution of A, C, and E influences on Musical Expertise as dependent on the level of lifetime amount of music practice.

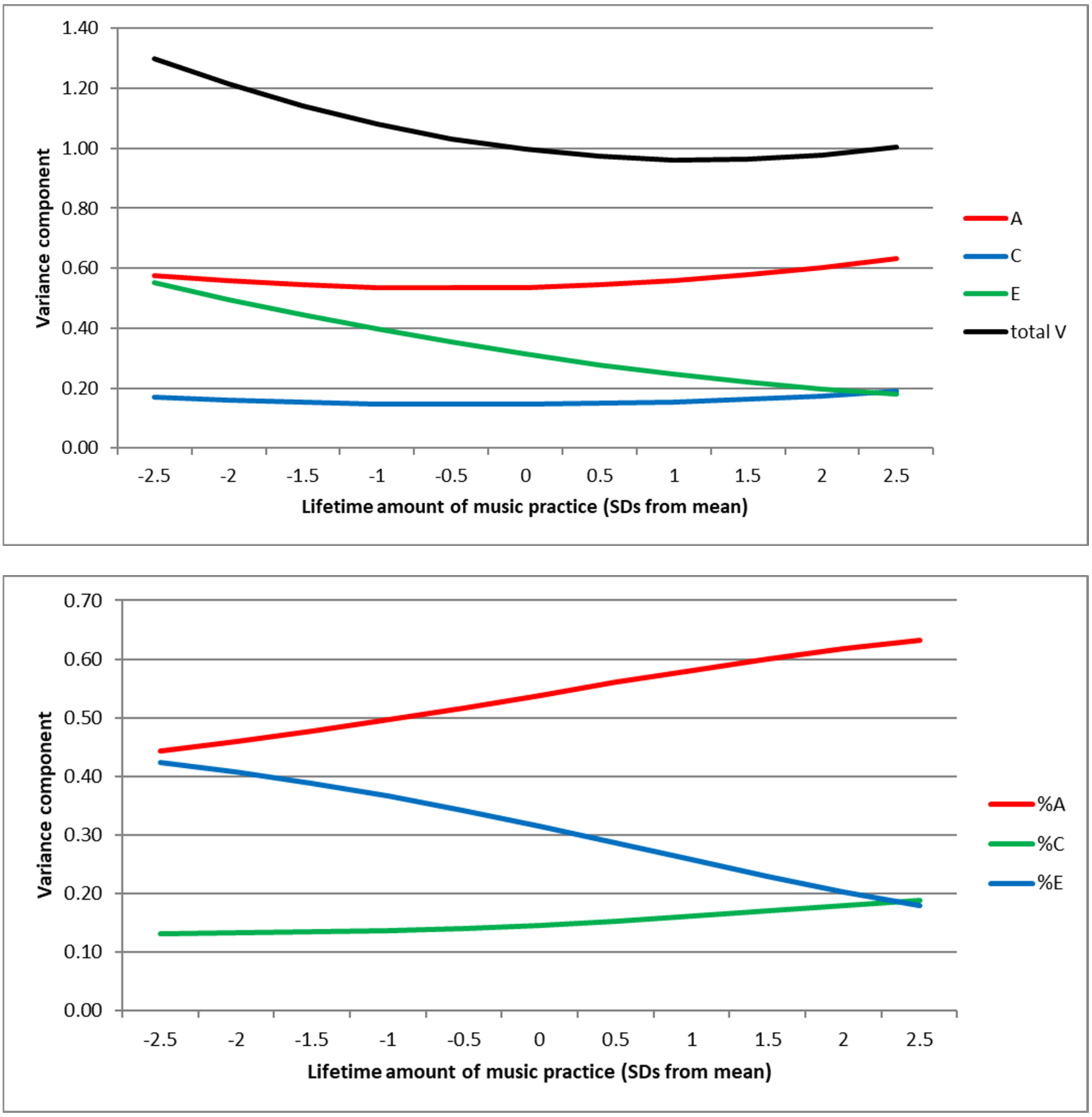

References

Carson, S., Peterson, J. B., & Higgins, D. M. (2005). Reliability, validity, and factor structure of the creative achievement questionnaire. Creative Research Journal, 17(1), 37-50.

Formann, A. K., & Piswanger, K. (1979). Wiener Matrizen Test [Vienna Matrices Test]: Ein Rasch-skalierter sprachfreier Intelligenztest. Weinheim: Beltz.

Madison, G. (2001). Variability in isochronous tapping: higher-order dependencies as a function of inter tap interval. J Exp Psychol: Hum Percept Perf, 27, 411-422.

Mosing, M.A., Verweij, K.J.H., Madison, G., & Ullén, F. (2016). The genetic architecture of correlations between perceptual timing, motor timing, and intelligence. Intelligence, 57: 33-40.
